# Serum Tumstatin and Vascular Endothelial Growth Factor Levels in Patients with Rosacea: A Cross-Sectional Case–Control Study

**DOI:** 10.3390/diagnostics16203347

**Published:** 2026-10-14

**Authors:** Mahir Dığış, Emel Güneş, Kısmet Kaya, Betül Demir, Süleyman Aydın

**Affiliations:** 1 Department of Dermatology and Venereology, Faculty of Medicine, Fırat University, 23119 Elazığ, Türkiye; 2 Department of Medical Biochemistry, Faculty of Medicine, Fırat University, 23119 Elazığ, Türkiye

**Keywords:** rosacea, vascular endothelial growth factor, tumstatin, angiogenesis, serum biomarkers, disease severity

## Abstract

**Background/Objectives:** Angiogenesis is a core feature of rosacea and vascular endothelial growth factor (VEGF) is upregulated in lesional skin, yet serum tumstatin, an endogenous angiogenesis inhibitor, has not been measured in rosacea. **Methods:** In this cross-sectional case–control study, serum VEGF and tumstatin were measured by enzyme-linked immunosorbent assay in 44 patients with rosacea and 44 age- and sex-comparable controls, with each analyte measured on a single plate. Results above the upper limit of quantification were treated as censored. Groups were compared with the Mann–Whitney U test and Tobit regression adjusted for age, sex and body mass index. **Results:** Both biomarkers were lower in rosacea: median VEGF 3.39 versus 5.50 ng/mL (*p* < 0.001; adjusted geometric mean ratio 0.71, 95% CI 0.58–0.86) and tumstatin 13.27 versus >20 ng/mL (*p* < 0.001; ratio 0.70, 95% CI 0.56–0.87). The analytes were very strongly correlated (ρ = 0.95), also within the measuring range (ρ = 0.91). Neither correlated with the Rosacea Area and Severity Index, disease duration or subtype, and discrimination was modest (area under the curve 0.72 and 0.70). **Conclusions:** In this preliminary single-centre study, serum VEGF and tumstatin were lower in patients with rosacea. Because the two analytes covaried to a degree that suggests a shared analytical factor, the samples were measured in singlicate, the kits were not validated in-house, many results exceeded the calibrated range, and VEGF concentrations (control median 5.50 ng/mL) exceeded published serum values from other immunoassays (≈0.04–0.23 ng/mL) more than tenfold, an analytical explanation cannot be excluded; the findings require confirmation with validated assays before any biological interpretation.

## 1. Introduction

Rosacea is a common chronic inflammatory disorder of the centrofacial skin, characterised by persistent erythema, flushing, telangiectasia, papules, pustules, phymatous change, and ocular involvement, and affects approximately 5.5% of adults [1]. Its pathophysiology is incompletely understood but is attributed to an interplay between innate and adaptive immune activation—with a proposed polarisation towards T-helper (Th)1 and Th17 responses—neurovascular dysregulation, and impaired epidermal barrier function [2,3]. A feature that distinguishes rosacea from purely inflammatory dermatoses is prominent vascular activation, and angiogenesis is now regarded as an integral rather than secondary component of its pathogenesis [4,5].

Vascular endothelial growth factor (VEGF), a potent mediator of endothelial proliferation, vascular permeability, and inflammatory amplification, occupies a central position in this vascular component [5]. Immunohistochemical studies consistently demonstrate increased VEGF activity in lesional skin: VEGF and its receptors are expressed on vascular endothelium and infiltrating inflammatory cells [6], and dermal VEGF staining is higher in lesional than in non-lesional skin [7]. Upstream, cathelicidin LL-37- and interleukin-33 (IL-33)-mediated innate immune signalling and mast-cell activation increase VEGF release, coupling recognised rosacea triggers to neovascularisation [8,9]. A VEGF +405C/G promoter polymorphism confers roughly 1.7- to 2.3-fold higher disease risk and greater erythematotelangiectatic severity [10], and cutaneous VEGF intensity decreases with effective treatment, consistent with a pathogenic and treatment-responsive role [11]. Non-invasive tape-stripping likewise identifies elevated lesional VEGF-α that correlates with disease severity [12].

In contrast to this tissue-level evidence, data on circulating VEGF in rosacea are sparse and inconsistent. Serum VEGF was reported to be approximately twofold higher than in controls among perimenopausal women with rosacea [13], serum VEGF did not differ significantly from controls in a subset of 20 patients in a genetic case–control study [10], and, in a multiplex study, tear-fluid VEGF was lower than in controls in cutaneous and ocular rosacea whereas no significant difference in serum VEGF was reported [14], indicating that VEGF alterations are compartment- and phenotype-dependent and that systemic concentrations may not mirror lesional activity. Circulating VEGF is elevated across many chronic inflammatory and autoimmune diseases, but with substantial heterogeneity attributable to disease subtype, sampling matrix, and assay conditions [15].

Pathological angiogenesis reflects an imbalance between pro-angiogenic drivers and endogenous inhibitors rather than an increase in VEGF alone [16]. Tumstatin—the non-collagenous (NC1) domain of the α3 chain of type IV collagen—suppresses endothelial proliferation and induces endothelial apoptosis through αvβ3 integrin-dependent signalling [17] and, together with other basement-membrane-derived fragments, restrains angiogenesis [18]. Tumstatin and VEGF are functionally linked at the endothelial cell surface, where tumstatin inhibits endothelial adhesion and survival on matrix-bound VEGF via integrin ligation [19]. Despite this relationship, circulating tumstatin—and the systemic balance between VEGF and anti-angiogenic type IV collagen fragments—has not been measured in rosacea.

Attempts to establish reliable systemic biomarkers of rosacea severity have produced mixed results: serum hypoxia-inducible factor-1α (HIF-1α) shows the most consistent association with severity [20], whereas markers such as endocan and endoglin do not reliably discriminate patients from controls [21], leaving the incremental value of circulating biomarkers over direct clinical assessment uncertain [22]. Age may further modulate the angiogenic response, as aged rosacea models show attenuated VEGF induction and reduced microvessel density [23]. On this basis, the present cross-sectional case–control study measured serum VEGF and tumstatin concentrations in patients with rosacea and healthy controls and evaluated their relationship with disease severity, clinical subtype, and age.

## 2. Materials and Methods

### 2.1. Study Design and Ethical Approval

This single-centre, cross-sectional case–control study was conducted at a university dermatology and venereology department; participants were enrolled between April and July 2026. The study protocol was approved by the institutional non-interventional research ethics committee (session date: 16 April 2026; session no: 2026/06-37) and was conducted in accordance with the Declaration of Helsinki. Written informed consent was obtained from all participants.

### 2.2. Study Population

A total of 88 participants were enrolled, including 44 patients with clinically diagnosed rosacea and 44 healthy control subjects. Rosacea was diagnosed based on characteristic clinical findings confirmed by dermatological examination. Clinical subtypes were recorded according to the 2002 National Rosacea Society (NRS) standard classification [24] as erythematotelangiectatic (*n* = 29), papulopustular (*n* = 15), and ocular (*n* = 2); no phymatous cases were observed. Each patient was assigned to one cutaneous subtype (erythematotelangiectatic or papulopustular), whereas ocular involvement was recorded as an additional, non-exclusive feature and was present in one patient with erythematotelangiectatic and one with papulopustular rosacea; the subtype counts therefore sum to 46. The phenotype-based approach recommended by the 2017 NRS update and the ROSacea COnsensus (ROSCO) panel [3,25] was not applied, which is acknowledged as a limitation. Demodex density was assessed by standardised skin surface biopsy, with a density exceeding 5 mites/cm^2^ regarded as positive; Demodex positivity was detected in 33 patients; Demodex density was not assessed in controls. Disease severity was graded using the Rosacea Area and Severity Index (RASI) [26] by a dermatologist who was blinded to the laboratory results. Control subjects were recruited from individuals attending the dermatology outpatient clinic for routine evaluation (naevus examination, cosmetic consultation or hair complaints) and had no history of rosacea or active inflammatory skin disease. Exclusion criteria for both groups included age under 18 years, pregnancy, systemic disease (diabetes mellitus, hypertension, cardiovascular disease, autoimmune disease, malignancy or thyroid disease), and current systemic medication use, including hormonal contraception, hormone replacement therapy, statins, antihypertensive and antiplatelet agents, and non-steroidal anti-inflammatory drugs. Patients who had used any oral antibiotic (including tetracyclines and macrolides), oral isotretinoin, a systemic glucocorticosteroid, immunosuppressant or biologic agent, or any topical rosacea treatment (including metronidazole, azelaic acid, ivermectin, brimonidine, oxymetazoline, retinoids, corticosteroids, calcineurin inhibitors and topical antibiotics), within the preceding three months were also excluded, as were patients who had undergone vascular laser, intense pulsed light or other dermatological or aesthetic procedures within the same period. Treatment history was verified from the medical history and hospital records. Menopausal status was not recorded.

### 2.3. Blood Sampling and Pre-Analytical Handling

Venous blood samples were collected from both patients and healthy controls at the study visit, in the morning after an overnight fast and under identical conditions for both groups. Serum was separated by centrifugation at 3000 rpm for 10 min, and aliquots were frozen immediately after separation and stored at −80 °C until analysis. All samples were assayed in a single batch on 14 July 2026, after completion of enrolment; the storage period therefore did not exceed three months, and each aliquot was thawed once for the assay. Clotting time before centrifugation, relative centrifugal force and haemolysis were not recorded, and complete blood counts (including platelet counts) were available for patients only. No residual serum remained after the assays.

### 2.4. Measurement of VEGF and Tumstatin

Serum VEGF concentrations were determined using a commercial enzyme-linked immunosorbent assay (ELISA) kit (YLBiont, Shanghai YL Biotech Co., Ltd., Shanghai, China; catalogue no. YLA1208Hu; analytical range 20–6000 ng/L [0.02–6.0 ng/mL]; sensitivity 10.42 ng/L; intra-assay coefficient of variation [CV] < 8%, inter-assay CV < 10%, as stated by the manufacturer). Serum tumstatin was measured using a commercially available ELISA kit (YLBiont; catalogue no. YLA4341Hu; analytical range 0.05–20 ng/mL; sensitivity 0.022 ng/mL; intra-assay CV < 8%, inter-assay CV < 10%). All assays were performed in the Department of Medical Biochemistry, Faculty of Medicine, Fırat University, according to the manufacturer’s instructions. For each analyte, all 88 samples (44 patients and 44 controls) were analysed on a single 96-well plate from one kit lot, in one run on the same day, by the same operator, who was blinded to group allocation; the remaining eight wells were used for the kit standards, so that the standard curve was run in singlicate; neither a separate blank (zero-standard) well nor an independent quality-control sample was documented. Patient and control samples were therefore always analysed on the same plate, so that between-plate variation could not contribute to the between-group comparison. Samples were loaded in two consecutive blocks (patients followed by controls), in the same order on both plates, and were measured in singlicate. Absorbance was measured using a BioTek Epoch2 microplate reader (BioTek Instruments, Winooski, VT, USA), and washing steps were performed with a BioTek ELx50 microplate washer (BioTek Instruments). Concentrations were calculated by the laboratory from the kit standard curve; the curve-fitting model was not documented and raw absorbance data were not retained. No in-house validation of the kits (dilution linearity, spike recovery, or cross-reactivity of the tumstatin antibody with intact type IV collagen) was performed; the absolute concentrations are therefore kit-specific and may not be comparable with values obtained with other immunoassays. Samples exceeding the upper limit of the analytical range were not re-assayed after dilution, because no residual serum remained; the corresponding concentrations were reported by the laboratory as values extrapolated beyond the highest calibrator (VEGF, 21/88 [23.9%], maximum 6.49 ng/mL; tumstatin, 30/88, maximum 24.90 ng/mL) or, for four tumstatin samples, as “>25.125 ng/mL” without a numerical value. Because extrapolated values have no defined accuracy, all results above the upper limit of quantification (ULOQ; VEGF, 21/88 [23.9%]; tumstatin, 34/88 [38.6%]) were treated as right-censored at the ULOQ in the primary analysis (Section 2.5); extrapolated values were used only in a supplementary analysis.

### 2.5. Statistical Analysis

Statistical analyses were performed using Python version 3.12.13 (Python Software Foundation, Wilmington, DE, USA; pandas, NumPy, SciPy, statsmodels, and scikit-learn). The primary objective, as stated in the ethics application, was to compare serum VEGF and tumstatin concentrations between patients with rosacea and controls; VEGF and tumstatin were therefore co-primary endpoints. All other analyses (age-stratified, diagnostic-performance, VEGF–tumstatin correlation and sensitivity analyses) were defined after data collection and are exploratory. No formal a priori sample-size calculation was performed; the sample size was determined by the number of eligible patients enrolled during the study period and by the capacity of a single 96-well plate per analyte. Normality was assessed using the Shapiro–Wilk test, and non-normal distributions were identified for VEGF and tumstatin in both groups (all *p* < 0.001). Non-parametric methods were therefore used for the primary comparisons. Results above the ULOQ were treated as right-censored at the ULOQ (Section 2.4): all such results were assigned the ULOQ value and therefore shared a common highest rank, so that the Mann–Whitney U test could be applied without assigning numerical values beyond the calibrated range (equivalent to Gehan’s generalised Wilcoxon test for data censored at a common limit). This approach was adopted during revision in place of the analysis of extrapolated values, and all analyses other than the supplementary analysis of extrapolated values used the censored data. Between-group comparisons of biomarker concentrations and continuous baseline variables were performed using the Mann–Whitney U test, with effect sizes expressed as the rank-biserial correlation and the common-language effect size; for baseline variables and for the supplementary analysis of extrapolated values, between-group differences were estimated with the Hodges–Lehmann estimator and 95% confidence intervals (CIs), an estimator that is not defined for censored data. Sex, smoking, and alcohol use were compared using the Pearson chi-square test, or Fisher’s exact test where expected cell counts were below 5. As VEGF and tumstatin were co-primary endpoints, *p* values were additionally reported after Bonferroni correction. Between-group differences on the concentration scale were estimated by Tobit (censored normal) regression of ln-transformed concentration, right-censored at ln(ULOQ), with group as the only predictor and with additional adjustment for age, sex and body mass index (BMI); the group coefficient was back-transformed and is reported as the ratio of geometric means (GMR, rosacea/control) with 95% CI. Associations between variables were evaluated using Spearman’s rank correlation coefficient; the VEGF–tumstatin correlation was additionally estimated after restriction to samples with both results within the measuring range, and plate-position effects were assessed as the Spearman correlation between each analyte and sample loading order within each group. Receiver operating characteristic (ROC) analysis was used to assess the discriminative performance of VEGF and tumstatin for distinguishing patients from controls, with results reported as area under the curve (AUC) and bootstrap 95% confidence intervals; the two AUCs were compared by bootstrap resampling of their difference. Optimal cut-offs were identified using the Youden index, and sensitivity and specificity at these cut-offs were additionally reported after bootstrap optimism correction. Effect modification by age was assessed post hoc by adding a group × age interaction term to a Tobit model of the ln-transformed marker, adjusted for sex and BMI. Age tertiles were defined by the 33.3rd and 66.7th percentiles of the pooled age distribution (cut-points 31 and 40 years); because age was recorded in whole years, tertile sizes were unequal (31, 30 and 27 participants), and no participant was aged 41 years. A supplementary analysis repeated the primary comparison using the extrapolated values as reported by the laboratory. Continuous variables are presented as median (interquartile range [IQR]). A two-sided *p* < 0.05 was considered statistically significant. *p* values below 0.001 are reported as *p* < 0.001.

This observational study is reported in accordance with the STROBE (Strengthening the Reporting of Observational Studies in Epidemiology) statement (Table S1).

### 2.6. Use of Generative Artificial Intelligence

DeepL (DeepL SE, Cologne, Germany) was used for language editing of the manuscript. ChatGPT 5.4 (OpenAI, San Francisco, CA, USA) was used to generate the graphical abstract submitted with the original version, which has been withdrawn from the revised submission. The authors take full responsibility for the content of the manuscript.

## 3. Results

### 3.1. Study Population and Baseline Characteristics

Eighty-eight participants were analysed (44 rosacea, 44 controls). The number of individuals screened for eligibility and the reasons for non-enrolment were not systematically recorded; all 88 enrolled participants were included in the analysis (participant flow in Figure S1). No participant was excluded after data cleaning and both biomarkers were available for all 88 participants (0% missing data for the primary outcomes). The groups did not differ significantly in any baseline continuous or categorical variable, and all Hodges–Lehmann 95% confidence intervals for the between-group location shift included zero (Table 1 and Table 2). The largest imbalance, body mass index (Hodges–Lehmann shift +1.3 kg/m^2^, 95% CI −0.20 to 2.80; *p* = 0.095), was carried forward as a covariate in the sensitivity analyses (Section 3.6).

### 3.2. Primary Outcome: Circulating VEGF and Tumstatin

**Figure 1:**
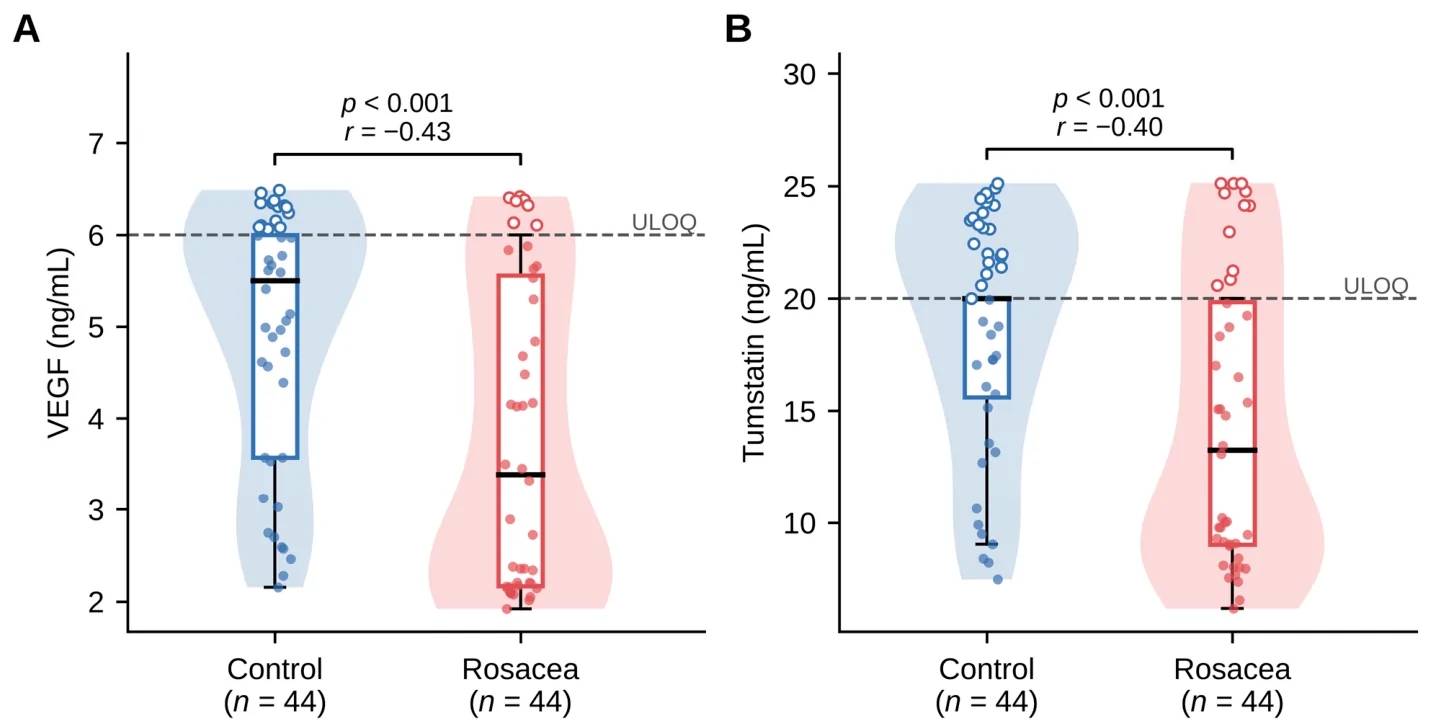
Serum VEGF (**A**) and tumstatin (**B**) concentrations in patients with rosacea and healthy controls. Violin shapes show the kernel density of the distribution, boxes show the median and interquartile range with whiskers extending to 1.5 × IQR, and individual participants are overlaid as jittered points. *p* values are from the Mann–Whitney U test with results above the upper limit of quantification (ULOQ) treated as censored, and *r* is the rank-biserial correlation. Dashed horizontal lines indicate the upper limit of the assay measuring range (6.0 ng/mL for VEGF, 20 ng/mL for tumstatin). Points above these lines (open circles) are extrapolated values (or, for four tumstatin results, values reported as >25.125 ng/mL) and are shown for display only. *n* = 44 per group. VEGF, vascular endothelial growth factor; IQR, interquartile range.

Both primary biomarkers were significantly lower in the rosacea group than in controls (Table 3, Figure 1).

Median serum VEGF was 3.39 ng/mL (IQR 2.17–5.56) in rosacea versus 5.50 ng/mL (IQR 3.57 to >6.00) in controls (Mann–Whitney U = 1386; *p* < 0.001; Bonferroni-adjusted *p* < 0.001). The effect size was moderate (rank-biserial correlation *r* = −0.432), and the common-language effect size (CLES) was 0.716, indicating that in 71.6% of randomly drawn control–patient pairs the control participant had the higher VEGF value. In Tobit regression, the geometric mean VEGF concentration in rosacea was 0.68 times that in controls (95% CI 0.56–0.84).

Median serum tumstatin was 13.27 ng/mL (IQR 9.02–19.85) in rosacea, whereas in controls the median lay above the ULOQ (>20.00 ng/mL; lower quartile 15.60 ng/mL) (U = 1360; *p* < 0.001; Bonferroni-adjusted *p* = 0.002; *r* = −0.405; CLES = 0.702; GMR 0.66, 95% CI 0.52–0.83).

Both comparisons remained significant after Bonferroni correction for the two primary endpoints (α = 0.025). Figure 1 displays the distributions as combined violin, box and individual-data-point plots; the dashed horizontal lines mark the upper limits of the assay measuring ranges (6.0 ng/mL for VEGF and 20 ng/mL for tumstatin), above which 21 VEGF and 34 tumstatin results fell; these are plotted at their extrapolated or reported values for display only (see Section 3.6).

### 3.3. Age-Stratified Post Hoc Analysis

**Figure 2:**
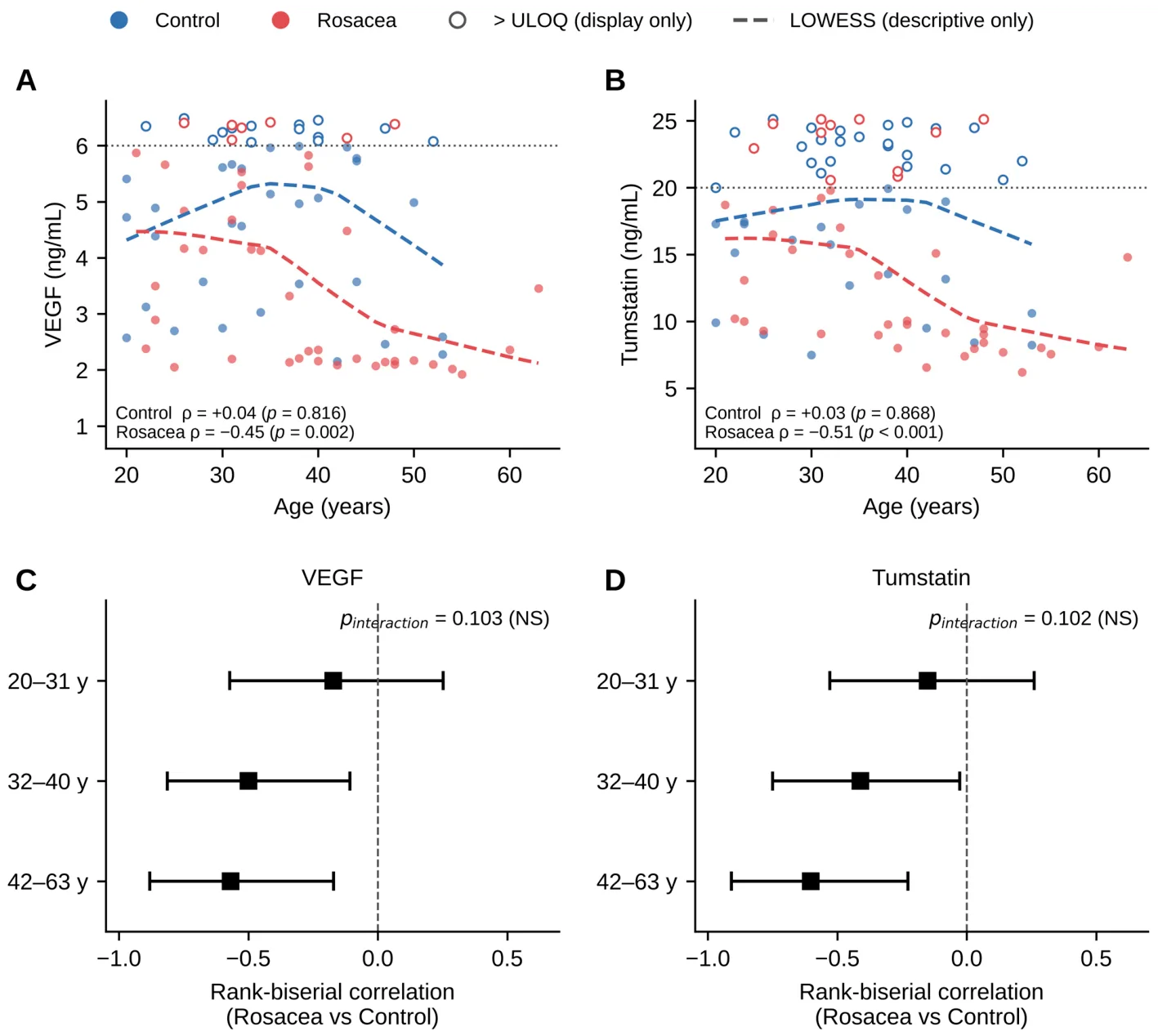
Age dependence of the group difference. (**A**,**B**) Serum VEGF (**A**) and tumstatin (**B**) plotted against age for controls (blue) and patients with rosacea (red); open circles denote results above the upper limit of quantification (dotted horizontal lines), plotted at their extrapolated or reported values for display only, with colour indicating group; dashed lines are LOWESS smoothers shown for description only and not used for inference. Within-group Spearman coefficients are inset. (**C**,**D**) Post hoc case–control rank-biserial correlations for VEGF (**C**) and tumstatin (**D**) within age tertiles; squares are point estimates and horizontal bars are bootstrap 95% confidence intervals. The vertical dashed line marks the null (*r* = 0). *p*_interaction_ is derived from a group × age interaction term in a Tobit model of the ln-transformed marker (censored at the upper limit of quantification), adjusted for sex and BMI (VEGF *p* = 0.103; tumstatin *p* = 0.102). Spearman coefficients and effect sizes are based on values censored at the upper limit of quantification. NS, not significant. LOWESS, locally weighted scatterplot smoothing; BMI, body mass index.

Because rosacea biology and angiogenic tone are both age-dependent, the group difference was examined across age strata post hoc (Figure 2, Table 4). Neither biomarker correlated with age within the control group (VEGF: Spearman ρ = +0.036, *p* = 0.816; tumstatin: ρ = +0.026, *p* = 0.868), whereas both declined significantly with age within the rosacea group (VEGF: ρ = −0.454, *p* = 0.002; tumstatin: ρ = −0.508, *p* < 0.001; Figure 2A,B). All within-group coefficients were calculated on the censored values; for VEGF in the rosacea group, the coefficient was virtually identical to that obtained with the extrapolated values (ρ = −0.4542 versus −0.4536), because the seven censored results were spread across the age range (26–48 years). The locally weighted scatterplot smoothing (LOWESS) curves are descriptive and were not used for inference.

Across age tertiles (T1: 20–31 years, *n* = 31; T2: 32–40, *n* = 30; T3: 42–63, *n* = 27) the case–control effect size showed a monotonic gradient for both biomarkers, negligible in T1 and moderate-to-large and significant in T2 and T3 (Table 4, Figure 2C,D). The group × age interaction nevertheless did not reach significance (VEGF *p*interaction = 0.103; tumstatin *p*interaction = 0.102). These subgroup analyses were not pre-specified, not corrected for multiplicity, and are underpowered within strata (11–17 participants per group per tertile); they are hypothesis-generating only.

### 3.4. Diagnostic Performance

**Figure 3:**
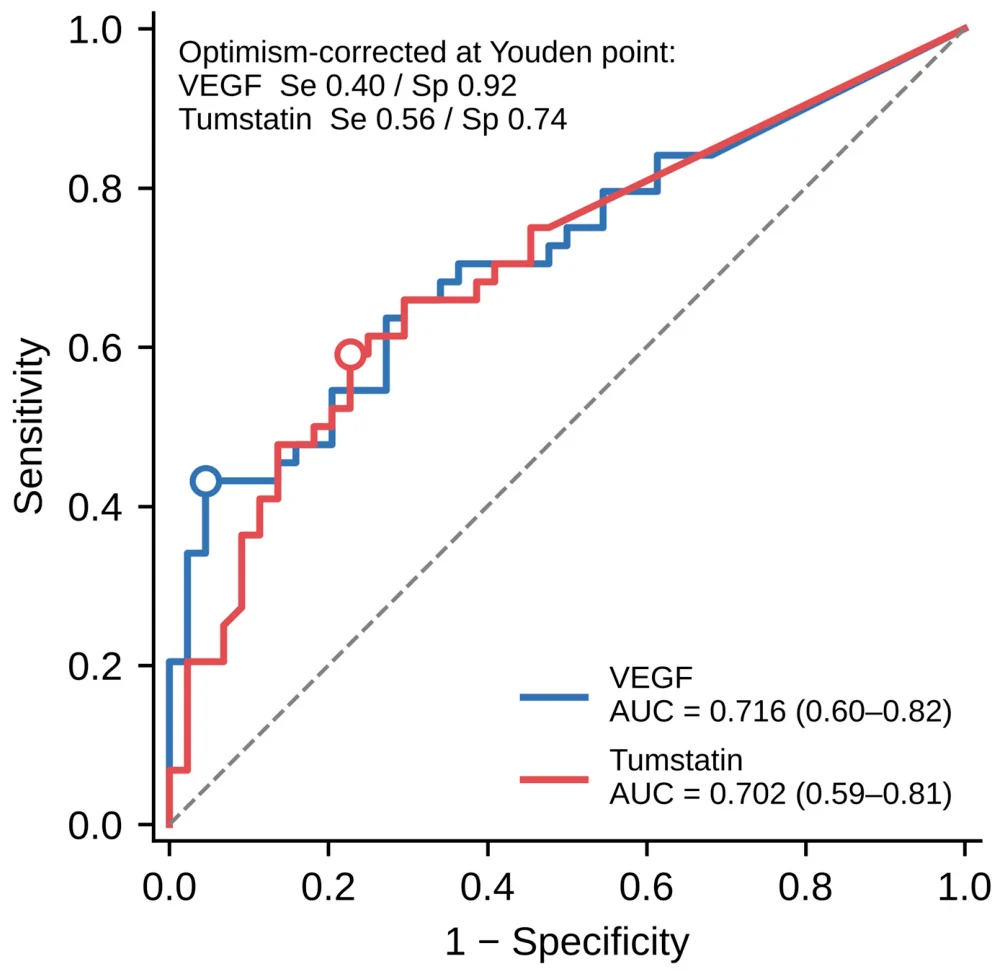
Receiver operating characteristic curves for serum VEGF (blue) and tumstatin (red) discriminating rosacea from control status. Circles mark the Youden-optimal operating point of each curve. The diagonal dashed line denotes chance performance. Areas under the curve are shown with bootstrap 95% confidence intervals. Optimism-corrected sensitivity and specificity at the Youden point are annotated at top left. *n* = 88 (44 cases, 44 controls).

Discrimination of rosacea from control status was assessed by ROC analysis (Table 5, Figure 3). VEGF yielded an AUC of 0.716 (bootstrap 95% CI 0.603–0.818) and tumstatin 0.702 (0.588–0.807); both confidence intervals excluded 0.50 but both upper bounds remained below 0.85, indicating discrimination that is statistically better than chance yet insufficient for stand-alone diagnostic use. The two AUCs did not differ significantly (bootstrap difference 0.013, 95% CI −0.025 to 0.060; *p* = 0.50). At the Youden-optimal cut-offs, VEGF behaved as a highly specific but insensitive rule-in marker and tumstatin as a more balanced but less specific one (Table 5). Because the cut-offs were derived and evaluated in the same dataset, the apparent operating characteristics are optimistically biased; bootstrap optimism correction lowered them further (Table 5, Figure 3). No external validation cohort was available.

### 3.5. Relationship Between VEGF and Tumstatin

**Figure 4:**
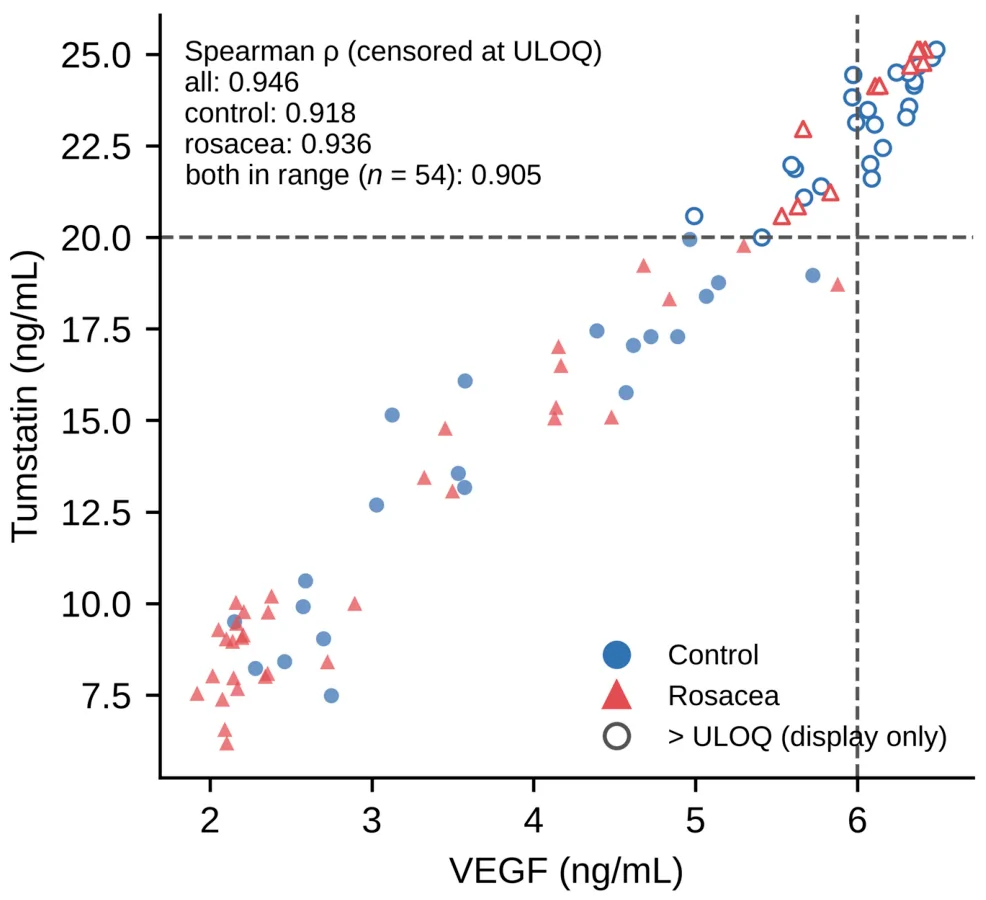
Relationship between serum VEGF and tumstatin concentrations. Controls are shown as blue circles and patients with rosacea as red triangles; open symbols denote samples with at least one result above the upper limit of quantification, with colour and shape indicating group; dashed lines indicate the upper limits of the assay measuring ranges, and points beyond them are extrapolated values (or, for four tumstatin results, values reported as >25.125 ng/mL) shown for display only. Spearman coefficients (values censored at the upper limit of quantification) for the full cohort and for each group, and for the 54 samples with both results within the measuring range, are inset. *n* = 88.

VEGF and tumstatin were very strongly and positively correlated across the whole cohort (Spearman ρ = 0.946 for censored values and 0.968 for extrapolated values; both *p* < 0.001; *n* = 88), with a similar magnitude within each group (controls ρ = 0.918; rosacea ρ = 0.936). The correlation persisted after restriction to the 54 samples in which both results lay within the measuring range (ρ = 0.905, *p* < 0.001; controls ρ = 0.930, *n* = 21; rosacea ρ = 0.874, *n* = 33) and after adjustment for group and sample loading order (partial ρ = 0.938); it is therefore not explained by the assay ceiling. Neither analyte was associated with loading order within either group (all |ρ| ≤ 0.07, all *p* ≥ 0.67), providing no evidence of a within-plate positional gradient. Figure 4 shows the relationship, with the assay upper limits indicated by dashed lines.

### 3.6. Sensitivity Analyses

Two potential sources of bias, the handling of results above the ULOQ and covariate imbalance, were addressed in exploratory sensitivity analyses (Table 6). Results above the ULOQ were more frequent among controls than patients (VEGF, 14 vs. 7; tumstatin, 23 vs. 11) and clustered concordantly across the two analytes: of the 12 samples with VEGF above 6.3 ng/mL, 11 also had tumstatin above 24 ng/mL, and four tumstatin results were reported only as >25.125 ng/mL, consistent with saturation of the standard curve rather than free measurement. Because ceiling values predominated in the group with the higher concentrations, any resulting compression would bias the observed group difference towards the null rather than create it. Analysis of the extrapolated values as reported gave similar results, as did Tobit regression with and without adjustment for age, BMI and sex (Table 6). Bonferroni correction across the two primary endpoints is reported in Table 3; subgroup, correlation and exploratory analyses (Section 3.3, Section 3.5 and Section 3.7) were not corrected, and their nominal *p* values should be interpreted accordingly.

### 3.7. Exploratory Analyses Within the Rosacea Group

Within the 44 patients with rosacea, neither biomarker was associated with disease severity or duration (Table 7). RASI score (median 10.50, IQR 5.75–13.83) did not correlate with VEGF (ρ = −0.151, *p* = 0.328) or tumstatin (ρ = −0.127, *p* = 0.410). Disease duration likewise showed no association (VEGF ρ = 0.190, *p* = 0.217; tumstatin ρ = 0.200, *p* = 0.193). Platelet counts, available for patients only (median 273.5 × 10^9^/L, IQR 242.3–310.5), were not correlated with VEGF (ρ = 0.082, *p* = 0.596) or tumstatin (ρ = 0.056, *p* = 0.716).

Neither Demodex status nor clinical subtype stratified VEGF or tumstatin levels: no significant difference was found by Demodex positivity (*n* = 33 vs. 11; VEGF *p* = 0.724; tumstatin *p* = 0.413), erythematotelangiectatic subtype (*n* = 29 vs. 15; *p* = 0.602 and 0.671) or papulopustular subtype (*n* = 15 vs. 29; *p* = 0.602 and 0.671). The phymatous subtype was absent from the cohort and ocular involvement was present in only 2 patients; neither could be tested.

These exploratory comparisons are underpowered, uncorrected for multiplicity, and negative results here should not be read as evidence of the absence of an association.

## 4. Discussion

In this cross-sectional case–control study, serum concentrations of both VEGF and tumstatin were lower in patients with rosacea than in controls. The effect sizes were moderate (rank-biserial *r* = −0.432 for VEGF and −0.405 for tumstatin) and persisted after Bonferroni correction, Tobit regression adjusted for age, body mass index and sex, and analysis of extrapolated rather than censored values. The two analytes were very strongly correlated, also within the measuring range, declined with age within the rosacea group only, and showed no association with disease severity, duration or clinical subtype; discrimination was modest (area under the curve 0.72 and 0.70). Because analytical and biological explanations cannot be separated with the present data, the findings are regarded as preliminary and are discussed with this constraint in view.

The direction of the VEGF finding is discordant with the dominant tissue-based narrative. Immunohistochemical and tape-stripping studies report increased VEGF in lesional rosacea skin [6,7,12], and serum VEGF was reported to be elevated in perimenopausal women with rosacea [13]. Other circulating data are limited and non-significant: in a subset of 20 patients and 20 controls, Hayran et al. found numerically lower serum VEGF in rosacea (median 179 versus 227 pg/mL; *p* = 0.176) and a trend towards higher serum VEGF receptor-1 (10 per group; *p* = 0.070) [10], and Topcu-Yilmaz et al., using a multiplex bead assay, found lower tear-fluid VEGF in patients with rosacea but no significant difference in serum VEGF [14]. The direction of the present finding is therefore consistent with these two small studies, although neither of them found a statistically significant difference in serum VEGF. Circulating VEGF is generally raised across chronic inflammatory and autoimmune diseases [15]. If the reduction observed here is biological, sequestration of VEGF within lesional skin and infiltrating inflammatory cells [6] and counter-regulatory mediators such as soluble VEGF receptor-1, which tended to be higher in rosacea [10] and is elevated in some inflammatory skin and connective-tissue diseases [27,28], are possible explanations; neither was tested in this study. An analytical explanation is at least as plausible, as discussed below.

To our knowledge, circulating tumstatin has not previously been measured in rosacea. Tumstatin, the NC1 domain of the α3 chain of type IV collagen, is an endogenous inhibitor of angiogenesis [16,17,18], and a simple pro-/anti-angiogenic imbalance model would predict reduced tumstatin together with increased VEGF. The concurrent reduction in both markers does not fit this model and, given the analytical considerations below, should not be interpreted mechanistically until it has been reproduced with independently validated assays.

The very strong correlation between VEGF and tumstatin (ρ = 0.95 for censored and 0.97 for extrapolated values) is the most important caveat of this study. It persisted among the 54 samples in which both results lay within the measuring range (ρ = 0.91) and is therefore not explained by saturation of the standard curves, contrary to our original interpretation. Plate-to-plate variation cannot account for it, because each analyte was measured on a single plate containing both groups, and neither analyte was associated with loading order within either group. Two proteins of different origin and regulation are not expected to covary to this degree in serum; in our view, a shared sample-level factor affecting both immunoassays, such as matrix effects, haemolysis, lipaemia, total protein, pipetting variation or non-specific binding, is the most plausible explanation, and circulating angiogenic biomarkers measured by ELISA are known to be vulnerable to pre-analytical and matrix effects [29,30]. These factors were not recorded, samples were measured in singlicate, raw absorbance data were not retained, and no residual serum was available for re-analysis, so this explanation could not be tested. Biological co-regulation, for example through the interaction of tumstatin with matrix-bound VEGF at the endothelial surface [19], cannot be excluded but is unlikely to produce a correlation of this magnitude. A shared sample-level factor would also affect the between-group comparison if its distribution differed between patients and controls; the lower concentrations of both markers in patients may therefore be partly analytical in origin.

Both biomarkers declined with age within the rosacea group (ρ = −0.454 for VEGF and −0.508 for tumstatin) but not within controls, and the case–control effect size increased across age tertiles. An age-dependent attenuation of the angiogenic response has been described in an aged LL-37 murine rosacea model [23], whereas circulating VEGF does not uniformly fall with age in the general population [15,31,32]. However, the group × age interaction was not significant (*p* = 0.103 for VEGF and 0.102 for tumstatin), the analyses were post hoc and underpowered, and the analytical caveats above apply equally; these observations are hypothesis-generating only.

Neither marker correlated with the Rosacea Area and Severity Index, disease duration, or clinical subtype, and the optimism-corrected operating characteristics (VEGF sensitivity 0.40, specificity 0.92; tumstatin sensitivity 0.56, specificity 0.74) fall short of stand-alone diagnostic utility. Local cutaneous VEGF-α measured by tape-stripping correlates with rosacea severity [12], whereas the systemic concentrations measured here did not. The result is congruent with the broader view that circulating biomarkers add limited value over direct clinical assessment of rosacea severity [22] and argues against routine diagnostic use of serum VEGF or tumstatin.

The strengths of this study include complete primary-outcome data, analysis of both groups on the same plate for each analyte by an operator blinded to group allocation, blinded severity scoring, and censoring-aware analyses with covariate adjustment. Its limitations are substantial. First, the analytical layer is weak: commercial kits were used without in-house validation (dilution linearity, spike recovery, or specificity of the tumstatin antibody for the α3(IV)NC1 fragment), samples were measured in singlicate, the standard curve was run in singlicate without a documented blank or quality-control sample (limiting the accuracy of the calibration), the curve-fitting model was not documented, raw absorbance data were not retained, a large proportion of results exceeded the highest calibrator and could not be re-assayed after dilution because no residual serum remained, and the absolute VEGF concentrations (control median 5.50 ng/mL) are more than an order of magnitude higher than the serum medians of approximately 0.04–0.23 ng/mL reported for rosacea and control groups with other immunoassays [10,14]. Second, pre-analytical variables relevant to serum VEGF, which is largely released from platelets during clotting [30], were incompletely documented: clotting time, centrifugal force and haemolysis were not recorded, and platelet counts were available for patients only. Third, samples were loaded in group blocks rather than randomised across the plate; although no positional gradient was detected, plate position cannot be fully separated from group. Fourth, the single-centre, cross-sectional design, the absence of systematic screening logs, the modest sample size without an a priori sample-size calculation, the underpowered and multiplicity-uncorrected subgroup analyses, and the absence of an external validation cohort for the ROC estimates limit inference. Fifth, menopausal status was not recorded, Demodex density was assessed in patients only, subtypes were classified according to the 2002 NRS scheme rather than the phenotype-based approach, and ocular (*n* = 2) and phymatous (*n* = 0) presentations were nearly absent. Sex was balanced between the groups and included as a covariate, but the study was not powered for sex-disaggregated analyses.

## 5. Conclusions

In this preliminary single-centre study, serum VEGF and tumstatin were both lower in patients with rosacea than in controls and were unrelated to clinical severity. Because the two analytes covaried to a degree that suggests a shared analytical factor, and because the assays were not validated in-house, were measured in singlicate and frequently exceeded the calibrated range, and because the absolute VEGF concentrations (control median 5.50 ng/mL) were more than an order of magnitude higher than published serum values obtained with other immunoassays (approximately 0.04–0.23 ng/mL [10,14]), an analytical explanation for these findings cannot be excluded, and no mechanistic conclusion is drawn. The data do not support serum VEGF or tumstatin as diagnostic biomarkers of rosacea. Confirmation requires independent cohorts with randomised plate layouts, duplicate measurements, dilution of high samples, documented pre-analytical handling and assays validated against reference methods, ideally paired with lesional measurements.

## Figures and Tables

**Table 1:** Baseline continuous characteristics of the study groups.

Variable	Control (*n* = 44)	Rosacea (*n* = 44)	*p*	*r*_rb_	HL Difference [95% CI]
Age, years	34.50 [29.75–40.50]	37.50 [31.00–46.25]	0.249	0.143	2.0 [−2.00, 7.00]
Height, cm	167.00 [162.00–176.25]	165.00 [160.00–173.00]	0.676	−0.052	−1.0 [−5.00, 3.00]
Weight, kg	70.00 [65.00–84.00]	73.00 [65.75–84.25]	0.295	0.130	3.0 [−2.00, 8.50]
BMI, kg/m^2^	25.15 [22.68–27.45]	26.25 [24.68–29.32]	0.095	0.207	1.3 [−0.20, 2.80]

Values are median [interquartile range]. *p* values are from the Mann–Whitney U test. *r*_rb_, rank-biserial correlation (effect size); HL difference, Hodges–Lehmann estimate of the median between-group difference (rosacea minus control) with its 95% confidence interval. BMI, body mass index. CI, confidence interval.

**Table 2:** Baseline categorical characteristics of the study groups.

Variable	Control (*n* = 44)	Rosacea (*n* = 44)	Test	Min. Expected	*p*
Male sex	14/44 (31.8)	13/44 (29.5)	Pearson χ^2^	13.5	1.000
Current smoking	14/44 (31.8)	14/44 (31.8)	Pearson χ^2^	14.0	1.000
Alcohol use	3/44 (6.8)	4/44 (9.1)	Fisher exact	3.5	1.000

Values are *n*/*N* (%). Pearson’s chi-square test was used when all expected cell counts were ≥5; Fisher’s exact test was used otherwise. Min. expected, smallest expected cell count in the contingency table.

**Table 3:** Primary outcome: serum VEGF and tumstatin concentrations by group.

Marker	Control (*n* = 44)	Rosacea (*n* = 44)	U	*p*	*p*_Bonf_	*r*_rb_	CLES	Values > ULOQ, *n* (C/R)
VEGF, ng/mL	5.50 [3.57 to >6.00]	3.39 [2.17–5.56]	1386.0	<0.001	<0.001	−0.432	0.716	14/7
Tumstatin, ng/mL	>20.00 [15.60 to >20.00]	13.27 [9.02–19.85]	1360.0	<0.001	0.002	−0.405	0.702	23/11

Values are median [interquartile range]; results above the upper limit of quantification (ULOQ; 6.0 ng/mL for VEGF and 20 ng/mL for tumstatin) were treated as right-censored at the ULOQ, and quantiles lying in the censored range are shown as >ULOQ. U, Mann–Whitney U statistic (control group); *p*_Bonf_, *p* value after Bonferroni correction for the two primary endpoints; *r*_rb_, rank-biserial correlation; CLES, common-language effect size (probability that a randomly selected control exceeds a randomly selected patient); C, control; R, rosacea. Results obtained with the extrapolated values are given in Section 3.6. VEGF, vascular endothelial growth factor.

**Table 4:** Post hoc case–control effect sizes within age tertiles.

Tertile	Age Range, y	*n* (C/R)	Control Median	Rosacea Median	*r*_rb_ [95% CI]	*p*
VEGF, ng/mL						
T1	20–31	17/14	4.89	4.42	−0.172 [−0.57, 0.25]	0.423
T2	32–40	16/14	5.98	4.14	−0.500 [−0.81, −0.11]	0.019
T3	42–63	11/16	4.99	2.17	−0.568 [−0.88, −0.17]	0.014
*p*_interaction_ (group × age) = 0.103						
Tumstatin, ng/mL						
T1	20–31	17/14	17.45	17.42	−0.151 [−0.53, 0.26]	0.474
T2	32–40	16/14	>20.00	16.05	−0.411 [−0.75, −0.03]	0.043
T3	42–63	11/16	18.97	8.26	−0.602 [−0.91, −0.23]	0.009
*p*_interaction_ (group × age) = 0.102						

*r*_rb_, rank-biserial correlation (rosacea vs. control) with bootstrap 95% confidence interval; *p* values are nominal and uncorrected for multiplicity. *p*_interaction_ refers to the group × age interaction term from a Tobit model of the ln-transformed marker (censored at the ULOQ) adjusted for sex and BMI, evaluated across the full cohort. Medians and effect sizes are based on values censored at the ULOQ; >20.00 denotes a median above the ULOQ. Tertile cut-points are the 33.3rd and 66.7th percentiles of the pooled age distribution. Analyses were not pre-specified and are underpowered within strata.

**Table 5:** Diagnostic performance of serum VEGF and tumstatin for rosacea.

Marker	AUC [95% CI]	Youden Cut-Off	Sensitivity	Specificity	J	Se_corr_	Sp_corr_
VEGF	0.716 [0.603, 0.818]	≤2.38 ng/mL	0.432	0.955	0.386	0.40	0.92
Tumstatin	0.702 [0.588, 0.807]	≤15.10 ng/mL	0.591	0.773	0.364	0.56	0.74

AUC, area under the receiver operating characteristic curve with bootstrap 95% confidence interval (2000 resamples). Cut-offs were selected by maximising the Youden index (J = sensitivity + specificity − 1). Apparent sensitivity and specificity are derived and evaluated in the same dataset; optimism-corrected values were obtained by bootstrap correction (1000 resamples). No external validation was performed. Se_corr_ and Sp_corr_, optimism-corrected sensitivity and specificity.

**Table 6:** Sensitivity analyses for the primary outcome.

Analysis	*n* (C/R)	Effect Size	*p*
VEGF			
Primary (censored at ULOQ)	44/44	*r*_rb_ = −0.432	<0.001
Extrapolated values	44/44	*r*_rb_ = −0.416; HL = −1.13 [−2.18, −0.41]	<0.001
Tobit, unadjusted	44/44	GMR = 0.68 [0.56, 0.84]	<0.001
Tobit, adjusted (age + BMI + sex)	44/44	GMR = 0.71 [0.58, 0.86]	<0.001
Tumstatin			
Primary (censored at ULOQ)	44/44	*r*_rb_ = −0.405	<0.001
Extrapolated values	44/44	*r*_rb_ = −0.376; HL = −4.61 [−8.00, −1.36]	0.002
Tobit, unadjusted	44/44	GMR = 0.66 [0.52, 0.83]	<0.001
Tobit, adjusted (age + BMI + sex)	44/44	GMR = 0.70 [0.56, 0.87]	0.001

The primary analysis treats results above the upper limit of quantification (ULOQ; 6.0 ng/mL for VEGF, 20 ng/mL for tumstatin) as right-censored at the ULOQ. The extrapolated-values analysis uses the concentrations reported by the laboratory, with the four tumstatin results reported as >25.125 ng/mL assigned that value. Tobit models are censored normal regressions of ln-transformed concentration. *r*_rb_, rank-biserial correlation; HL, Hodges–Lehmann median difference (rosacea minus control, ng/mL) with 95% confidence interval; GMR, ratio of geometric means (rosacea/control) with 95% confidence interval. BMI, body mass index.

**Table 7:** Exploratory analyses within the rosacea group (*n* = 44).

Analysis	*n*	Effect Size	*p*
Correlation with clinical measures			
VEGF vs. RASI score	44	ρ = −0.151	0.328
Tumstatin vs. RASI score	44	ρ = −0.127	0.410
VEGF vs. disease duration	44	ρ = 0.190	0.217
Tumstatin vs. disease duration	44	ρ = 0.200	0.193
VEGF vs. platelet count	44	ρ = 0.082	0.596
Tumstatin vs. platelet count	44	ρ = 0.056	0.716
VEGF/tumstatin by Demodex status and clinical subtype (present/absent)			
Demodex positivity	33/11	—	0.724/0.413
Erythematotelangiectatic	29/15	—	0.602/0.671
Papulopustular	15/29	—	0.602/0.671
Phymatous	0/44	—	NT
Ocular involvement	2/42	—	NT

Correlations are Spearman’s ρ; subtype comparisons are Mann–Whitney U tests of VEGF and tumstatin concentrations (*p* values given as VEGF/tumstatin); all analyses use values censored at the upper limit of quantification. Platelet counts were available for patients only. RASI, Rosacea Area and Severity Index. All *p* values are nominal and uncorrected for multiplicity. NT, not testable.

## Data Availability

The data supporting the conclusions of this article are not publicly available because the informed consent form signed by the participants states that their data will not be shared with third parties. A de-identified minimal dataset was provided to the editorial office for verification during peer review, and the data are available from the corresponding author on reasonable request, within the limits of the participants’ consent.

## Supplementary Materials

The following supporting information can be downloaded at: https://www.mdpi.com/article/10.3390/diagnostics16203347/s1, Figure S1: Participant flow diagram; Table S1: STROBE checklist for case–control studies.

## Abbreviations

The following abbreviations are used in this manuscript:

AUC	Area under the receiver operating characteristic curve
BMI	Body mass index
CI	Confidence interval
CLES	Common-language effect size
CV	Coefficient of variation
ELISA	Enzyme-linked immunosorbent assay
GMR	Geometric mean ratio
HIF-1α	Hypoxia-inducible factor-1α
HL	Hodges–Lehmann
IL-33	Interleukin-33
IQR	Interquartile range
LOWESS	Locally weighted scatterplot smoothing
NC1	Non-collagenous 1 (domain)
NRS	National Rosacea Society
RASI	Rosacea Area and Severity Index
ROC	Receiver operating characteristic
ROSCO	ROSacea COnsensus
STROBE	Strengthening the Reporting of Observational Studies in Epidemiology
Th	T-helper
ULOQ	Upper limit of quantification
VEGF	Vascular endothelial growth factor
