# Supplementary material for: Serum Tumstatin and Vascular Endothelial Growth Factor Levels in Patients with Rosacea: A Cross-Sectional Case–Control Study
Source: Diagnostics (Basel). 2026 Oct 14;16(20):3347. doi: 10.3390/diagnostics16203347 (PMC13703348; doi:10.3390/diagnostics16203347)
Supplement: Supplementary file 1 [file diagnostics-16-03347-s001.zip › diagnostics-4591817-supplementary.pdf]

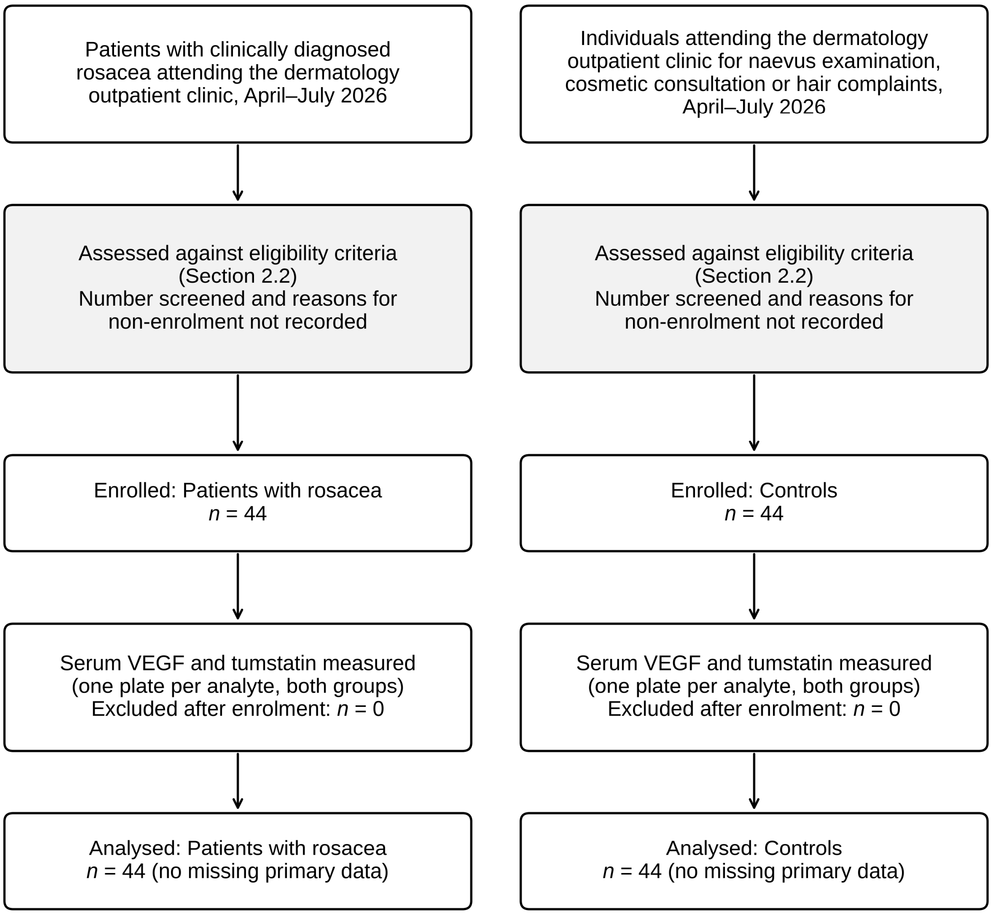

Figure S1. Participant flow diagram.

Table S1. STROBE checklist for case–control studies.

Section/topic	Item	Recommendation	Reported in (revised manuscript)
Title and abstract	1	(a) Indicate the study design with a commonly used term in the title or abstract; (b) provide an informative and balanced summary	Title; Abstract
Background/rationale	2	Explain the scientific background and rationale	Introduction, paragraphs 1–5
Objectives	3	State specific objectives, including any prespecified hypotheses	Introduction, last paragraph; Section 2.5 (primary objective)
Study design	4	Present key elements of study design early in the paper	Section 2.1
Setting	5	Describe the setting, locations and relevant dates, including periods of recruitment, exposure, follow-up and data collection	Sections 2.1 (enrolment April–July 2026), 2.3 (sampling, assay date 14 July 2026)
Participants	6	(a) Eligibility criteria, sources and methods of case ascertainment and control selection; rationale for the choice of cases and controls; (b) matching criteria	Section 2.2; groups not individually matched (age and sex comparable, Tables 1–2)
Variables	7	Clearly define all outcomes, exposures, predictors, potential confounders and effect modifiers	Sections 2.2, 2.4, 2.5
Data sources/measurement	8	Sources of data and details of methods of assessment; comparability of assessment methods if there is more than one group	Sections 2.3, 2.4 (both groups on the same plate per analyte; operator blinded)
Bias	9	Describe any efforts to address potential sources of bias	Sections 2.2 (blinded RASI scoring), 2.4 (blinded operator, single plate), 2.5 (censoring, Tobit adjustment); Section 4 (limitations)
Study size	10	Explain how the study size was arrived at	Section 2.5 (no a priori calculation; enrolment period and plate capacity)
Quantitative variables	11	Explain how quantitative variables were handled; describe groupings and why	Section 2.5 (ln transformation, censoring at ULOQ, age tertile cut-points)
Statistical methods	12	(a) All statistical methods including confounding control; (b) subgroups and interactions; (c) missing data; (d) matching; (e) sensitivity analyses	Section 2.5; (c) no missing primary data (Section 3.1); (d) not applicable; (e) Section 3.6, Table 6
Participants	13	(a) Numbers at each stage; (b) reasons for non-participation; (c) flow diagram	Section 3.1; Figure S1; numbers screened and reasons for non-enrolment were not recorded (Section 4)
Descriptive data	14	(a) Characteristics of participants and information on exposures and potential confounders; (b) number with missing data	Tables 1–2; Section 2.2; Section 3.1
Outcome data	15	Report numbers in each exposure category, or summary measures of exposure	Table 3; Figure 1
Main results	16	(a) Unadjusted and confounder-adjusted estimates and their precision; which confounders and why; (b) category boundaries for continuous variables; (c) absolute risk (if relevant)	(a) Tables 3 and 6; (b) Section 2.5 and Table 4; (c) not applicable
Other analyses	17	Other analyses done — subgroups, interactions, sensitivity analyses	Sections 3.3–3.7; Tables 4–7; Figures 2–4
Key results	18	Summarise key results with reference to study objectives	Section 4, paragraph 1
Limitations	19	Discuss limitations, including sources of potential bias or imprecision; direction and magnitude of any potential bias	Section 4, limitations paragraph; correlation paragraph
Interpretation	20	Cautious overall interpretation considering objectives, limitations, multiplicity and similar studies	Sections 4 and 5
Generalisability	21	Discuss the generalisability (external validity) of	Section 4, limitations paragraph

		the study results	
Funding	22	Source of funding and role of funders	Funding statement (no external funding)

Section and table/figure references are given instead of line numbers because line numbering will change during production.
